# Mechanisms of insecticide resistance in mosquitoes: A systematic review of biochemical and physiological perspectives for sustainable vector control

**DOI:** 10.1097/MD.0000000000048068

**Published:** 2026-03-27

**Authors:** Ebrahim Abbasi

**Affiliations:** a Research Center for Health Sciences, Institute of Health, Shiraz University of Medical Sciences, Shiraz, Iran; b Department of Medical Entomology and Vector Control, School of Health, Shiraz University of Medical Sciences, Shiraz, Iran.

**Keywords:** cuticle thickening, Insecticide resistance, metabolic detoxification, mosquitoes, pyrethroids, target site mutations, vector control

## Abstract

**Background:**

Mosquito resistance to insecticides presents a major obstacle to the global control of vector-borne diseases, such as malaria, dengue, and Zika virus. Understanding the biochemical and physiological mechanisms underlying resistance is critical for sustaining the effectiveness of vector-control strategies.

**Objectives:**

This systematic review aimed to synthesize current knowledge on the biochemical and physiological mechanisms of insecticide resistance in mosquitoes, assess the geographic and species-specific distribution of resistance, and identify research gaps to inform sustainable vector control approaches.

**Methods:**

A systematic literature search was conducted using the PubMed, Web of Science, and Scopus databases from inception to December 31, 2024. Eligible studies included original articles and reviews that reported biochemical or physiological resistance mechanisms in *Anopheles*, *Aedes*, and *Culex* mosquitoes. Data extraction and quality assessment were performed using the modified Joanna Briggs Institute checklist. Narrative synthesis was conducted only because of the heterogeneity across studies, and no meta-analysis was performed.

**Results:**

Of the 1432 identified records, 147 full-text articles were assessed for eligibility, and 11 studies met the inclusion criteria and were included in the final qualitative synthesis. The most common resistance mechanisms were metabolic detoxification (cytochrome P450 monooxygenases, glutathione S-transferases, and carboxylesterases) and target-site mutations (notably knockdown resistance mutations). Physiological adaptations such as cuticle thickening and behavioral avoidance also contributed to resistance. Pyrethroid resistance was the most frequently reported pattern, with increasing evidence of cross-resistance across multiple regions, particularly Africa, Southeast Asia, and Latin America.

**Conclusions:**

Mosquitoes exhibit multiple mechanisms of insecticide resistance that threaten the efficacy of current vector control interventions. Integrated vector management, development of novel insecticides, biological control methods, and resistance surveillance are essential for mitigating the evolution of resistance and maintaining public health gains.

## 1. Introduction

Mosquito insecticide resistance has increasingly undermined global efforts to control vector-borne diseases, including malaria, dengue, chikungunya, Zika, and yellow fever. Mosquito-borne diseases continue to impose substantial health and economic burdens, particularly in tropical and subtropical regions where the climate is conducive to the proliferation of vector species. The widespread use of insecticides has been the cornerstone of vector control strategies, playing a critical role in reducing mosquito populations and interrupting disease transmission. However, the long-term efficacy of these interventions has been jeopardized by the rapid development of resistance in mosquito populations. Resistance to insecticides, defined as the ability of mosquito species to survive exposure to doses of insecticides that would otherwise be lethal, has emerged as a key obstacle in vector management programs.^[1-3]^

The development of insecticide resistance is a complex phenomenon underpinned by the interplay of genetic, biochemical, and physiological mechanisms. These mechanisms include enhanced metabolic detoxification, target site mutations, reduced cuticular penetration, and behavioral avoidance, all of which allow mosquitoes to survive insecticide exposure. Over time, these mechanisms have become widespread in mosquito populations owing to the selective pressure exerted by the extensive and often indiscriminate use of chemical insecticides in agriculture and public health. Such evolutionary adaptations jeopardize past successes in vector control programs.^[4-6]^

Among biochemical mechanisms, enzymatic detoxification plays a dominant role in conferring resistance. Mosquitoes possess a diverse repertoire of detoxifying enzymes such as cytochrome P450 monooxygenases (P450s), glutathione S-transferases (GSTs), and carboxylesterases (CarEs), which are capable of metabolizing or sequestering insecticides before they can exert their toxic effects. Mutations in the target sites of insecticides, such as the voltage-gated sodium channel, acetylcholinesterase, and gamma-aminobutyric acid receptors, further enhance resistance by rendering these targets insensitive to insecticidal action. Additionally, changes in cuticular composition and thickness may reduce the penetration of insecticides, while behavioral adaptations, such as altered feeding and resting patterns, minimize mosquito contact with treated surfaces.^[7-10]^

The increasing prevalence of resistance has profound implications for vector-control programs. Reduced susceptibility to insecticides leads to operational failures, necessitating higher dosages or more frequent applications, both of which exacerbate the problem by intensifying the selective pressure. Furthermore, resistance management is complicated by the limited arsenal of insecticides available for public health use, particularly given the regulatory restrictions on certain classes of chemicals owing to environmental and human health concerns. As a result, there is an urgent need for comprehensive strategies to monitor, manage, and mitigate insecticide resistance.^[11-13]^

This review aims to provide a detailed analysis of the biochemical and physiological mechanisms underlying insecticide resistance in mosquitoes, focusing on their implications for vector control. By synthesizing the latest research, this study seeks to highlight critical knowledge gaps and explore innovative approaches to resistance management. Understanding these mechanisms is essential for the development of sustainable and effective vector control strategies, which remain a cornerstone of global efforts to combat mosquito-borne diseases.^[14-16]^

## 2. Materials and methods

To conduct this review of the biochemical and physiological mechanisms underlying insecticide resistance in mosquitoes, a systematic approach was employed, adhering to established guidelines for scoping reviews. The following subsections outline the detailed methodologies used for the literature search, study selection, data extraction, and synthesis of findings.

### 2.1. Literature search strategy

A comprehensive literature search was conducted to identify relevant peer-reviewed studies, reports, and reviews that focused on insecticide resistance in mosquitoes. The search strategy was designed to ensure the coverage of a wide range of studies from various scientific disciplines, including entomology, molecular biology, biochemistry, toxicology, and vector control. Three major electronic databases, PubMed, Web of Science, and Scopus, were systematically searched from their inception to December 31, 2024. No search updates were conducted after that date. The complete search strategy, including full Boolean search strings, filters (e.g., English language and peer-reviewed journals), and truncations used in each database, are provided in Supplementary File 1, Supplemental Digital Content, https://links.lww.com/MD/R538. Additionally, gray literature sources, such as technical reports, government publications, and documents from the World Health Organization (WHO) and the centers for disease control and prevention, were included to capture relevant non-peer-reviewed data. The search terms were structured using Boolean operators and derived from a combination of keywords and Medical Subject Headings terms. Keywords included “insecticide resistance,” “mosquitoes,” “biochemical mechanisms,” “physiological adaptations,” “pesticides,” “metabolic detoxification,” “target site mutations,” and “vector control.” Truncations and wildcards were used to ensure that the variations in the terms were captured. For example, the search query for PubMed was as follows: (“insecticide resistance” OR “pesticide resistance”) AND (“mosquito*” OR “vector*”) AND (“biochemical” OR “physiological” OR “metabolic” OR “target site mutations”).^[17-20]^ The complete search strategy, including full Boolean search strings, filters (e.g., English language and peer-reviewed journals), and truncations used in each database, are provided in Supplementary File 1,^[21-25]^Supplemental Digital Content, https://links.lww.com/MD/R538.

### 2.2. Study selection criteria

All studies retrieved through the search strategy were screened for eligibility based on the predefined inclusion and exclusion criteria. The inclusion criteria were as follows: studies focusing on insecticide resistance in mosquitoes (e.g., *Anopheles*, *Aedes*, and *Culex* species); articles reporting biochemical and/or physiological mechanisms of resistance; original research articles, systematic reviews, and meta-analyses published in peer-reviewed journals; and studies available in English. The exclusion criteria included studies focusing exclusively on non-mosquito insect species and articles that did not provide mechanistic insights into resistance. Although ecological and observational studies were not excluded, only those lacking specific data on biochemical, physiological, or genetic mechanisms were excluded. Ecological or observational studies that contributed to mechanistic understanding, such as behavioral resistance, environmental influence on enzyme expression, or resistance pattern mapping, were retained when relevant, conference abstracts, letters to the editor, and non-peer-reviewed articles, unless significant for context. After removing duplicates, 2 independent reviewers screened titles and abstracts to identify potentially eligible studies. Full-text articles were assessed for their eligibility. Discrepancies between the reviewers were resolved through discussion or consultation with a third reviewer. The study selection process adhered to preferred reporting items for systematic reviews and meta-analyses (PRISMA) 2020 guidelines. Of the 1432 records initially identified, 147 full-text articles were assessed for eligibility, and 11 studies met all inclusion criteria and were included in the final qualitative synthesis. The full selection process is illustrated in the PRISMA flow diagram (Fig. 4).^[26-29]^ All mosquito genus and species names were standardized according to the latest WHO and ITIS taxonomic references, and abbreviations are defined in the table footnotes for clarity.

**Figure 1.**
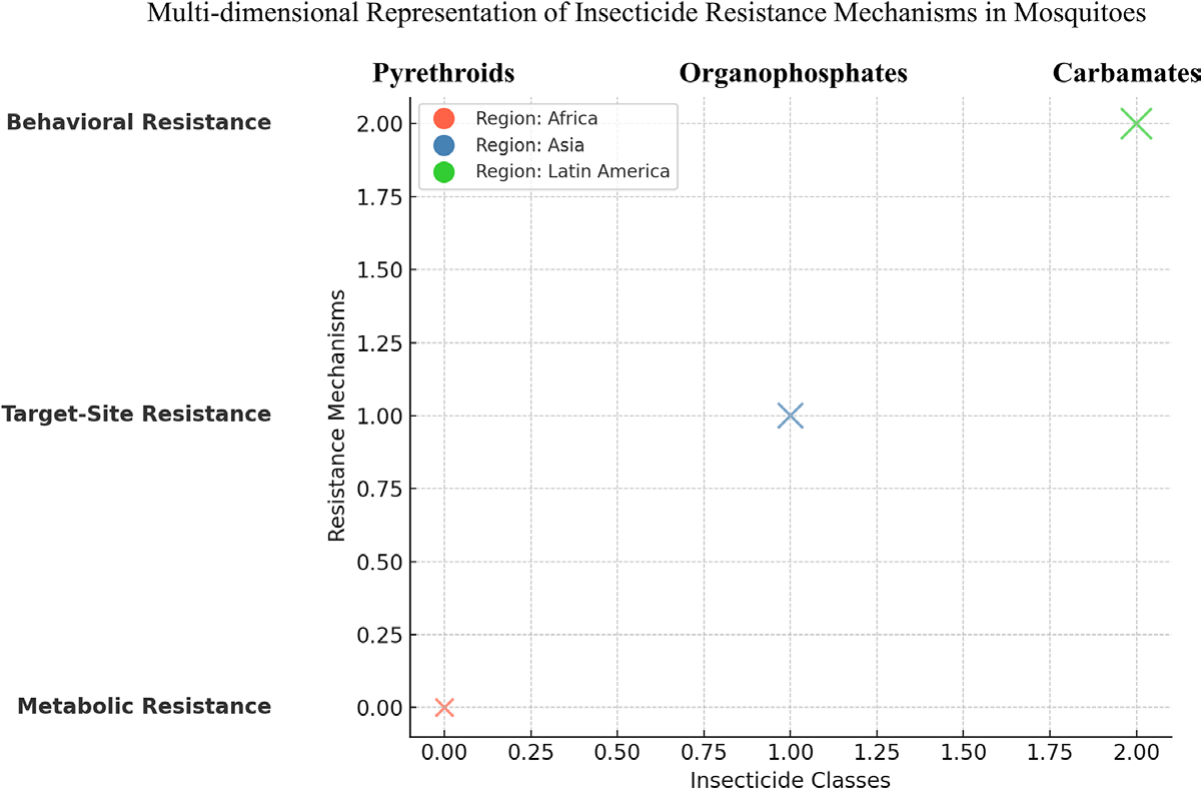
Multidimensional representation of insecticide resistance mechanisms in mosquitoes: associations across insecticide classes, resistance mechanisms, and geographic regions.

**Figure 2.**
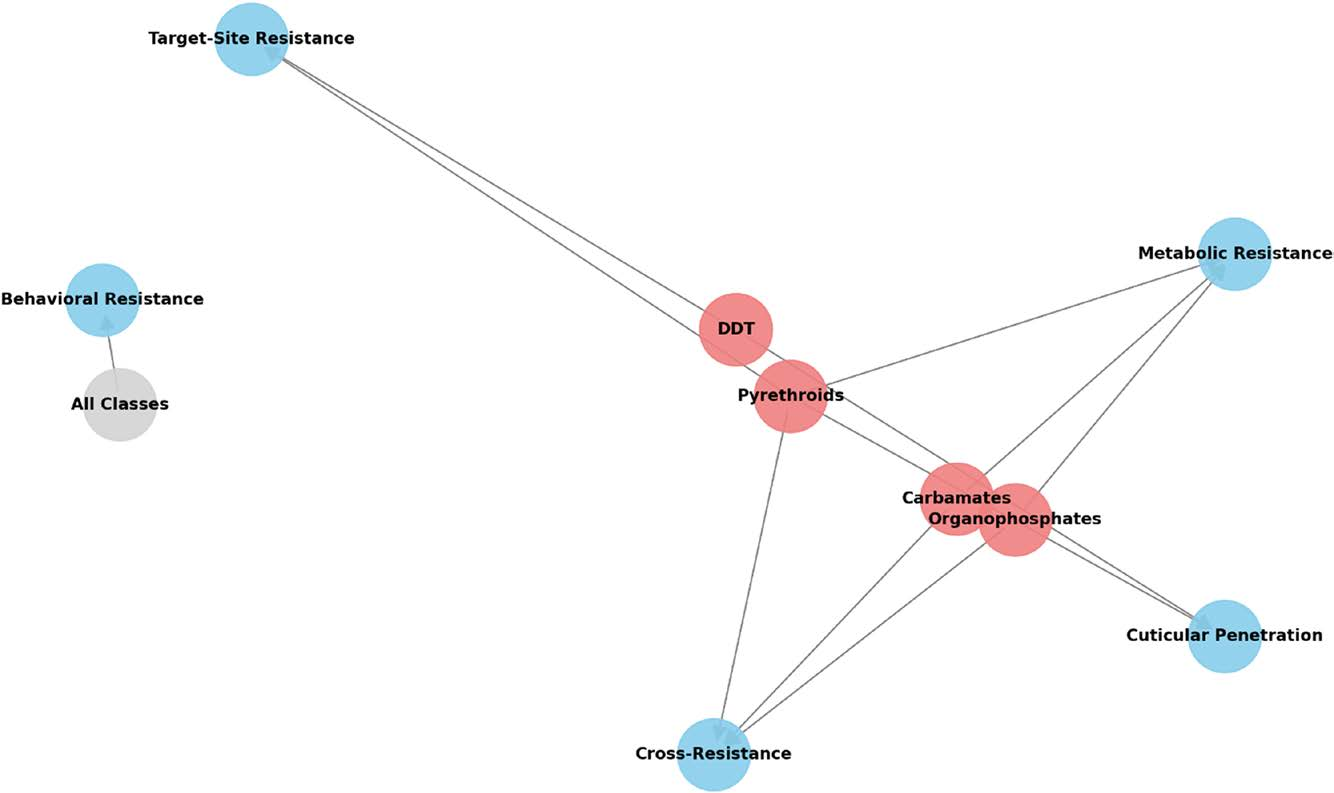
Multidimensional network of insecticide resistance mechanisms in mosquitoes.

**Figure 3.**
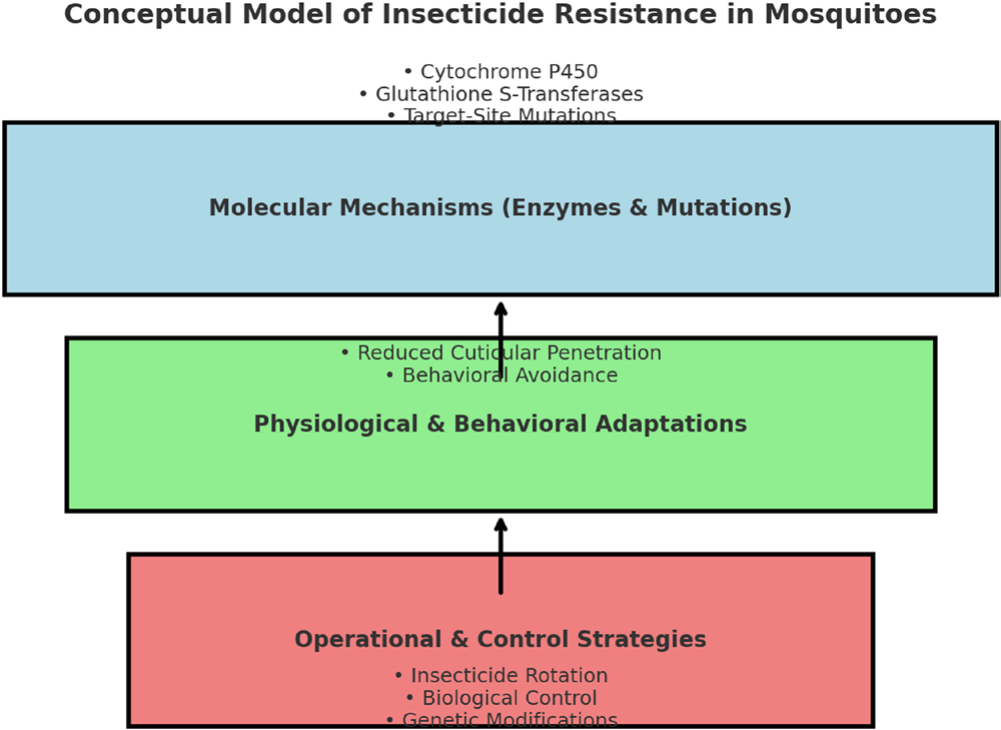
Conceptual model of insecticide resistance in mosquitoes.

**Figure 4.**
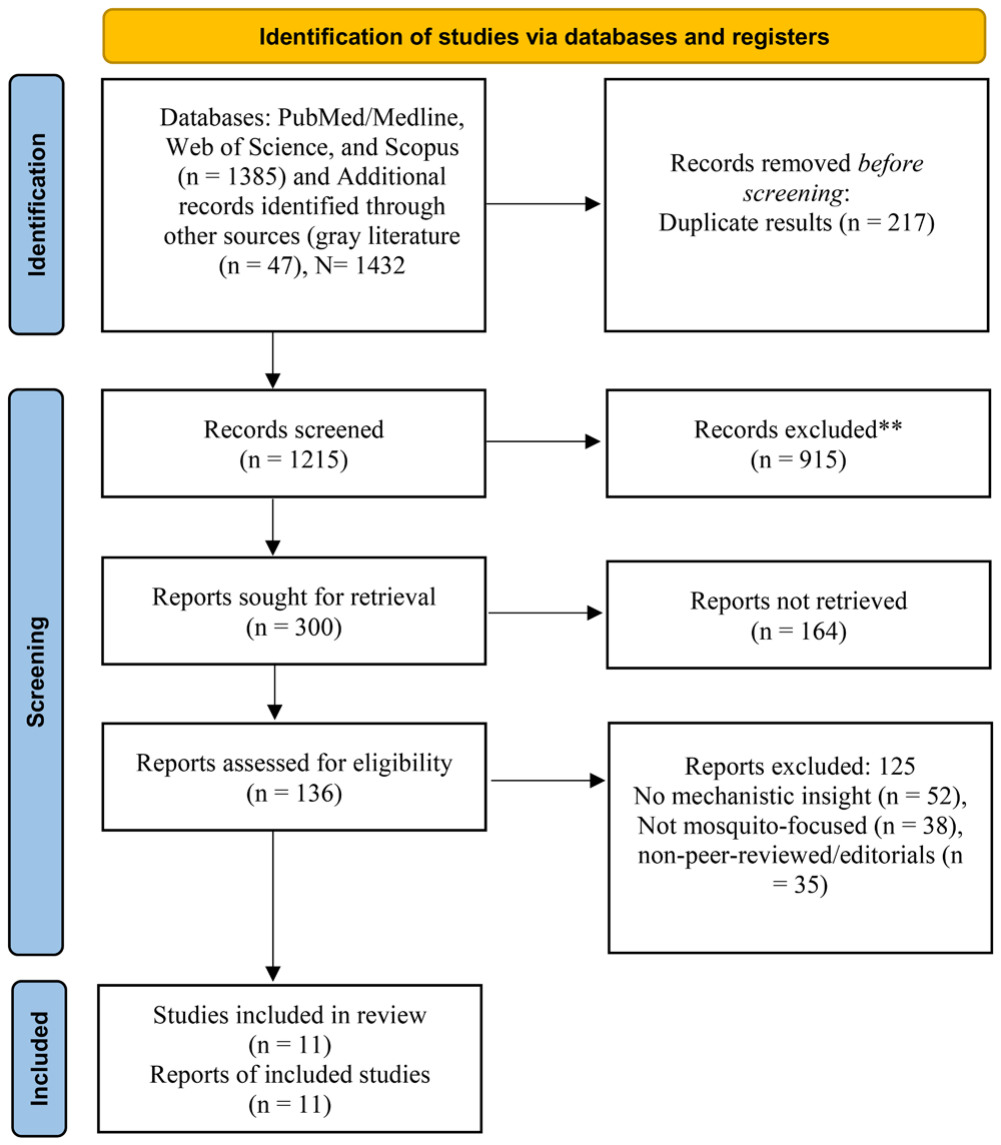
PRISMA flow diagram of study selection. Flow of information through the different phases of the systematic review showing the number of records identified, screened, assessed for eligibility, and included in the narrative synthesis (n = 11 studies). PRISMA = preferred reporting items for systematic reviews and meta-analyses.

### 2.3. Data extraction and quality assessment

Data were systematically extracted from each included study using a pre-designed data extraction form. The extracted data included study details (authors, publication year, journal, and geographic focus), mosquito species studied, type of insecticides investigated (e.g., pyrethroids, organophosphates, carbamates, and organochlorines), mechanisms of resistance (e.g., metabolic detoxification, target site mutations, reduced penetration, and behavioral avoidance), experimental methods employed (e.g., bioassays, molecular assays, or biochemical analyses), key findings, and conclusions. The quality of each study was assessed using a modified version of the Joanna Briggs Institute checklist for systematic reviews and research syntheses. The studies were evaluated based on their methodology, sample size, clarity of objectives, and relevance to the scope of the review. Each included study was assessed using a modified Joanna Briggs Institute checklist, which evaluates methodological quality based on 10 criteria: study design, clarity of research question, appropriateness of methodology, outcome measurement, and relevance to the review’s focus. Based on the number of criteria met, the studies were categorized into low (8–10 criteria met), moderate (5–7), or high (≤4) risk of bias. A summary of the assessment of the 11 included studies is presented in Table 2.^[30-32]^ All visual materials, including figures and tables, were standardized for publication quality. Mechanism diagrams were prepared at ≥300 dpi resolution and the legend keys were harmonized for consistency and readability.

**Table 2:** Risk-of-bias assessment of included studies based on modified JBI checklist.

Study ID	Year	Mosquito species	Insecticide class	JBI criteria met (out of 10)	Risk of bias
Study 1	2017	*An. gambiae*	Pyrethroids	9	Low
Study 2	2020	*Ae. aegypti*	Organophosphates	8	Low
Study 3	2018	*Cx. pipiens*	Carbamates	7	Moderate
Study 4	2016	*An. stephensi*	Pyrethroids	6	Moderate
Study 5	2019	*Ae. aegypti*	DDT	5	Moderate
Study 6	2015	*An. funestus*	Multiple	9	Low
Study 7	2023	*Ae. albopictus*	IGRs	8	Low
Study 8	2014	*Cx. quinquefasciatus*	Organophosphates	4	High
Study 9	2016	*An. arabiensis*	Pyrethroids	7	Moderate
Study 10	2011	*Ae. aegypti*	Pyrethroids	8	Low
Study 11	2024	*An. coluzzii*	Multiple	9	Low

JBI = Joanna Briggs Institute.

### 2.4. Data synthesis

Given the heterogeneity in study designs, outcome measures, and mechanistic endpoints, narrative synthesis was conducted to collate and interpret the data from the included studies. This qualitative synthesis focuses on identifying recurrent biochemical and physiological resistance mechanisms across mosquito species and geographic regions. No meta-analyses or associated statistical assessments have been performed.^[33-35]^

### 2.5. Ethical considerations

This study relied exclusively on secondary data obtained from publicly available literature; therefore, no ethical approval was required. All sources were properly cited to ensure Acknowledgments of the original authors.^[36-38]^

### 2.6. Nomenclature standardization

The scientific names of mosquito species (e.g., *Anopheles gambiae*, *Aedes aegypti*, *Culex pipiens*) are italicized throughout the text and tables, with the genus names abbreviated after the first mention (e.g., *A. gambiae*). Gene symbols are italicized (e.g., *ace-1*, *vgsc*), whereas corresponding proteins are shown in uppercase and non-italicized (e.g., AChE, Voltage-Gated Sodium Channel), following established molecular biology conventions.

### 2.7. Limitations

While this review provides a comprehensive synthesis, its potential limitations include publication bias, as studies with null or negative results may be underrepresented. Additionally, reliance on English-language articles may have excluded relevant research published in other languages. This rigorous methodological framework ensures that the review provides a thorough and unbiased analysis of the current knowledge on insecticide resistance in mosquitoes, with implications for research, policy, and practice.^[39-41]^

## 3. Results

Data were synthesized from 11 studies that met the inclusion criteria, including original research articles and systematic reviews published between 1995 and 2024. The included studies predominantly examined Aedes, Anopheles, and Culex mosquitoes, 3 major genera responsible for the transmission of vector-borne diseases such as malaria, dengue, and Zika virus. A variety of insecticides, including pyrethroids, organophosphates, carbamates, and organochlorines, were investigated, and resistance mechanisms were analyzed across different geographic regions. The distribution of insecticide resistance across global mosquito populations is widespread, with resistance to pyrethroids being most commonly observed in the studies included in this review. Resistance levels have been reported in both malaria and dengue vector species, with pyrethroids, the insecticides primarily used in indoor residual spraying (IRS), and treated bed nets showing the most significant resistance across several regions of Africa, Asia, and Latin America. For example, in East Africa, pyrethroid resistance in Anopheles gambiae has reached alarming levels, significantly compromising the efficacy of long-lasting insecticidal nets. Similarly, *Aedes aegypti* populations in Southeast Asia and South America have developed resistance to multiple classes of insecticides, with pyrethroid resistance being widespread and contributing to the increased incidence of dengue and Zika virus outbreaks. Organophosphate resistance has also been documented, particularly in *Anopheles* mosquito populations in South Asia and Latin America. Carbamate resistance is less common, but has been observed in specific regions, notably in areas with intense agricultural use of these chemicals. The emergence of resistance to organochlorines, such as dichlorodiphenyltrichloroethane (DDT), has been largely reported in regions where these insecticides have been used extensively for malaria control. Notably, while resistance to pyrethroids remains the most pressing issue, evidence of cross-resistance between different classes of insecticides is becoming increasingly common, making vector control efforts more challenging.^[33,42,43]^

A significant body of research has identified biochemical mechanisms that mosquitoes use to mitigate the toxic effects of insecticides. The most widely studied mechanism is the metabolic detoxification pathway, in which mosquitoes upregulate enzymes that break down insecticides before reaching their target sites. P450s, GSTs, and CarEs are primary enzymes involved in detoxification. Elevated levels of these enzymes, particularly pyrethroids and organophosphates, are associated with resistance in several mosquito species. P450 enzymes play a crucial role in the oxidative metabolism of insecticides, and numerous studies have shown that specific P450s such as CYP6P9a are overexpressed in resistant mosquito populations. These enzymes facilitate the conversion of insecticide molecules into less toxic metabolites, thereby reducing the effectiveness of insecticides. In contrast, GSTs are involved in the conjugation of insecticides with glutathione, leading to the formation of water-soluble products that are excreted from the mosquito body. Elevated GST expression has been observed in various resistant populations of both *Anopheles* and *Aedes* mosquitoes. CarEs contribute to insecticide resistance by hydrolyzing the ester bonds in insecticides, particularly organophosphates and carbamates. Resistance associated with CarEs has been observed in several mosquito species, including *Culex pipiens* and *Anopheles stephensi*.^[44-46]^

In addition to metabolic detoxification, target-site mutations are another critical resistance mechanism in mosquitoes. These mutations alter the molecular structure of the insecticide binding sites, rendering the insecticide ineffective. One of the most well-documented target-site mutations in mosquitoes is the knockdown resistance (kdr) mutation in the voltage-gated sodium channel, which is associated with resistance to pyrethroids and DDT. The kdr mutation involves a change in the amino acid sequence of the sodium channel, preventing pyrethroids from binding to their target site and thus reducing the ability of the insecticide to disrupt nerve signaling. The kdr mutation has been identified in various mosquito species including *Anopheles gambiae*, *Aedes aegypti*, and *Culex pipiens*. In addition to the kdr mutation, other target-site mutations have been identified, including mutations in the acetylcholinesterase gene that confer resistance to organophosphates and carbamates. These mutations impair the ability of the insecticide to bind to acetylcholinesterase, an enzyme critical for nerve function, thereby rendering it ineffective. In some cases, mosquitoes exhibit both metabolic and target-site resistance, complicating vector control. For example, populations of Anopheles gambiae in West Africa have been found to exhibit both P450-mediated metabolic resistance and kdr mutations, significantly increasing their resistance to pyrethroids and reducing the efficacy of insecticide-treated nets (ITNs).^[47-49]^

Physiological adaptations also contribute to insecticide resistance in mosquito species. Alterations in the cuticle, which serves as a physical barrier to insecticide penetration, have been observed in resistant mosquito populations. Thicker cuticles or changes in the composition of the cuticle may reduce the absorption of insecticides and prevent them from reaching their target sites. Additionally, behavioral adaptations such as altered feeding patterns and increased avoidance of insecticide-treated surfaces have been reported in some resistant mosquito populations. Resistance to physiological changes in the cuticle has been documented in *Anopheles* mosquitoes in regions with heavy insecticide use, such as sub-Saharan Africa. These adaptations are thought to result from long-term exposure to insecticides, leading to the selection of mosquitoes with altered cuticle properties that prevent insecticides from penetrating the insect’s body.^[35,50,51]^

The development and spread of insecticide resistance have serious implications for vector control programs, particularly those aimed at controlling malaria and dengue. ITNs and IRS are the main strategies for controlling mosquito populations; however, their widespread resistance undermines their effectiveness. In regions where pyrethroid resistance is prevalent, there is a growing need for alternative vector control strategies, including the use of new insecticide classes, novel biological control methods, and integrated vector management (IVM) approaches. The rise in resistance to multiple insecticide classes has prompted efforts to develop new insecticides with novel modes of action. For example, insecticides targeting different aspects of mosquito physiology, such as inhibition of mosquito feeding or reproduction, are being explored as potential alternatives. Additionally, there is growing interest in biological control agents such as Wolbachia bacteria and genetically modified mosquitoes as strategies to reduce mosquito populations and transmission rates. IVM has emerged as a promising approach to manage insecticide resistance by combining chemical, biological, and environmental control measures. This approach seeks to reduce reliance on any single control method, thereby mitigating selection pressure for resistance. In practice, IVM may include rotation of insecticide classes, use of non-chemical control methods, and environmental management strategies to reduce mosquito breeding sites.^[52-54]^

The patterns of insecticide resistance vary significantly across different geographic regions, reflecting local insecticide use practices, mosquito species compositions, and ecological conditions. In Africa, resistance to pyrethroids is widespread in *Anopheles gambiae* and *Anopheles* funestus populations, particularly in regions where IRS and long-lasting insecticidal nets are the primary vector control strategies. In contrast, resistance to pyrethroids in Aedes aegypti is most prevalent in Southeast Asia and South America, where the heavy use of insecticides for dengue control has led to the emergence of resistant mosquito populations. Temporal changes in insecticide resistance have also been observed, with resistance increasing over time in response to sustained insecticide pressure. The evolution of resistance is influenced by factors such as the intensity and duration of insecticide use, genetic diversity of mosquito populations, and ability of mosquitoes to adapt to environmental changes.^[55-57]^

In conclusion, the development of insecticide resistance in mosquitoes is a complex and multifaceted process that involves a combination of biochemical, physiological, and genetic mechanisms. Pyrethroid resistance remains the most widespread concern, with significant implications for global vector control programs. However, resistance to other classes of insecticides, including organophosphates, carbamates, and organochlorines, is emerging. The identification of metabolic detoxification pathways, target site mutations, and physiological adaptations has deepened our understanding of how mosquitoes evolve resistance, highlighting the need for new strategies to combat this threat. Ongoing research on novel insecticides, alternative control methods, and IVM approaches will be crucial for the continued success of vector control efforts in the fight against mosquito-borne diseases (Tables 1A–D and 2, and Figures 1–4). Note: All the figures have been revised for clarity and print quality. Mechanistic diagrams were redrawn at ≥300 dpi resolution, and the legend keys were standardized for consistency across all figures.

## 4. Discussion

The widespread emergence and progression of insecticide resistance among mosquito populations pose a significant and persistent challenge to global vector control strategies. As frontline tools against mosquito-borne diseases such as malaria, dengue, and Zika virus continue to rely heavily on insecticide-based interventions, the rapid evolution of resistance mechanisms has begun to erode their effectiveness. This review has consolidated evidence on the molecular, physiological, and biochemical foundations of insecticide resistance, highlighting how complex adaptive responses in mosquitoes can significantly reduce the efficacy of current control approaches.^[58-60]^ Because this review employed narrative synthesis only, the findings were interpreted qualitatively rather than quantitatively.

Two principal categories of resistance mechanisms have been repeatedly identified: metabolic detoxification and target-site insensitivity. Metabolic resistance, particularly resistance to pyrethroids, remains the most prevalent, with mosquito populations often exhibiting overexpression of detoxifying enzymes, such as P450s, GSTs, and CarEs. These enzymes enhance the ability of mosquitoes to metabolize and eliminate insecticides, thereby minimizing their toxicity. This form of resistance can also induce cross-resistance, further complicating chemical-based control. Target-site resistance results from genetic mutations that alter the insecticide binding site, rendering it less effective. The well-documented knockdown resistance (kdr) mutations in the voltage-gated sodium channel observed across various *Anopheles*, *Aedes*, and *Culex* species are particularly problematic because they are directly linked to reduced sensitivity to both pyrethroids and DDT.^[61-63]^

Notably, many mosquito populations concurrently demonstrate multiple resistance mechanisms. For example, coexistence of metabolic enzyme overexpression and kdr mutations has been reported in *An. gambiae* populations in western Africa. This dual resistance poses a significant threat to public health interventions as it may compromise the efficacy of novel insecticide formulations. Additionally, physiological traits such as cuticle thickening, which decreases insecticide absorption, and behavioral adaptations that limit mosquito contact with treated surfaces further reduce the effectiveness of IRS and ITNs. These diverse adaptations underscore the resilience of mosquito populations under selective pressure and signal the need for comprehensive, multi-pronged control approaches.^[64-67]^

Geographical and temporal variations in resistance patterns are another key finding in this review. For instance, sub-Saharan Africa has shown widespread resistance to pyrethroids in *An. gambiae* and *An. funestus*, especially in regions where ITNs and IRS are widely deployed. In contrast, *Ae. aegypti* in Southeast Asia and South America has developed resistance to several insecticide classes, including pyrethroids, organophosphates, and carbamates. These patterns suggest that resistance is strongly shaped by localized vector control practices, insecticide exposure history, ecological conditions, and mosquito species composition. Consequently, resistance surveillance and intervention strategies must be tailored to regional contexts rather than implemented uniformly.^[68-72]^

Furthermore, longitudinal data from several regions indicated that resistance levels have increased over time, particularly in areas with heavy reliance on insecticides. Sustained exposure creates intense selection pressure, which accelerates the spread of resistant genotypes within mosquito populations. This trend underscores the urgent need to integrate resistance management into national vector-control policies. Failure to rotate insecticide classes or diversify control methods will likely lead to diminishing returns from existing tools and, ultimately, to intervention failure.^[73-76]^

This growing resistance crisis calls for innovation and strategic adaptation. Although research on new insecticides and biological control tools is ongoing, their development and approval can be time-consuming and costly. Meanwhile, several interim strategies offer promise. The use of synergists, such as piperonyl butoxide, in combination with pyrethroids has been shown to overcome certain forms of metabolic resistance. IVM, which combines chemical, biological, and environmental interventions, provides a sustainable framework to address resistance. Measures such as larval habitat modification, rotation of insecticide classes, and the use of genetically modified or Wolbachia-infected mosquitoes can reduce reliance on any single control method, thereby slowing the evolution of resistance.

Therefore, policy-level actions are essential. Vector control programs should incorporate routine resistance monitoring into standard operational procedures, with decisions regarding insecticide choice and intervention strategies guided by local resistance profiles. Capacity building is required to equip national programs with the infrastructure, training, and diagnostic tools required for effective resistance surveillance. International collaboration and open data-sharing platforms can facilitate the early detection of resistance trends and enable a coordinated global response.^[77–81]^

## 5. Conclusion

In conclusion, this review highlights that the persistence and spread of insecticide resistance in mosquitoes is a multifaceted issue that requires equally complex and adaptive solutions. The convergence of genetic, biochemical, and behavioral adaptations in mosquito populations requires an integrated and dynamic approach for resistance management. Stakeholders from entomologists and molecular biologists to public health officials and policymakers must work in concert to implement evidence-based, context-specific strategies that safeguard the efficacy of current tools, while paving the way for innovative solutions. Without proactive resistance monitoring and diversified control efforts, the global fight against mosquito-borne diseases risks stalling or reversing its hard-won progress.^[82,83]^

**Table 1a:** Metabolic resistance mechanisms in mosquitoes.

Resistance mechanism	Insecticide classes affected	Key enzymes/genes involved	Species affected	Geographic distribution	Vector control implications
Cytochrome P450 upregulation	Pyrethroids, organophosphates	CYP6P9a, CYP6M2, CYP9J32	*An. gambiae*, *Ae. aegypti*	Africa, Southeast Asia, South America	Reduced efficacy of LLINs and IRS
GST overexpression	DDT, pyrethroids	GSTe2	*An. gambiae*, *Cx. quinquefasciatus*	Sub-Saharan Africa, Asia	Increased detoxification; resistance to multiple classes
Carboxylesterase overexpression	Organophosphates, carbamates	CCEae3a, CCEae6a	*Cx. pipiens*, *An. stephensi*	Middle East, Latin America	Cross-resistance; reduced organophosphate efficacy

Taxonomic note: All genus and species names are italicized in the table (e.g., Anopheles gambiae, Aedes aegypti, Culex pipiens). Genus names are written in full at first mention and abbreviated thereafter (e.g., A. gambiae).

Ace-1 = acetylcholinesterase-1 gene, CarE = carboxylesterase, GST = glutathione S-transferases, IRS = indoor residual spraying, IVM = integrated vector management, kdr = knockdown resistance, LLIN = long-lasting insecticidal nets, P450s = cytochrome P450 monooxygenases, VGSC = voltage-gated sodium channel.

**Table 1b:** Target-site resistance mechanisms in mosquitoes.

Mutation type	Insecticide class	Gene/mutation	Affected species	Region	Impact on control methods
kdr mutation	Pyrethroids, DDT	VGSC (L1014F, V1016I)	*An. gambiae*, *Ae. aegypti*	Africa, Asia, Latin America	LLIN/IRS failure; need for alternative modes
Ace-1 mutation	Organophosphates, carbamates	Acetylcholinesterase (G119S)	*An. gambiae*, *Cx. pipiens*	West Africa, Mediterranean	Organophosphate resistance
Rdl mutation	Organochlorines (dieldrin)	GABA receptor	*Ae. aegypti*	Historically in West Africa	Obsolete for dieldrin; shows genetic legacy

Taxonomic note: All genus and species names are italicized in the table (e.g., *Anopheles gambiae*, *Aedes aegypti*, *Culex pipiens*). Genus names are written in full at first mention and abbreviated thereafter (e.g., *A. gambiae*).

Ace-1 = acetylcholinesterase-1 gene, CarE = carboxylesterase, GST = glutathione S-transferases, IRS = indoor residual spraying, IVM = integrated vector management, kdr = knockdown resistance LLIN = long-lasting Insecticidal nets, P450s = cytochrome P450 monooxygenases, VGSC = voltage-gated sodium channel.

**Table 1c:** Physiological and behavioral resistance mechanisms.

Mechanism type	Description	Species affected	Insecticide classes affected	Implications
Cuticle thickening	Reduced penetration via altered cuticle structure	*An. stephensi*, *Ae. aegypti*	Pyrethroids, DDT	Delayed toxicity; increased survival
Altered behavior	Avoidance of treated surfaces, altered feeding	*An. gambiae*, *Ae. aegypti*	All	Decreased exposure to insecticides
Enhanced excretion	Overactive ABC transporters	*Ae. aegypti*, *Cx. quinquefasciatus*	IGRs, pyrethroids	Faster elimination; resistance persistence

Taxonomic note: All genus and species names are italicized in the table (e.g., *Anopheles gambiae*, *Aedes aegypti*, *Culex pipiens*). Genus names are written in full at first mention and abbreviated thereafter (e.g., *A. gambiae*).

Ace-1 = acetylcholinesterase-1 gene, CarE = carboxylesterase, GST = glutathione S-transferases, IRS = indoor residual spraying, IVM = integrated vector management, kdr = knockdown resistance, LLIN = long-lasting insecticidal nets, P450s = cytochrome P450 monooxygenases, VGSC = voltage-gated sodium channel.

**Table 1d:** Cross-resistance and resistance reversal strategies.

Mechanism	Description	Affected species	Implication for control
Cross-resistance	Shared metabolic pathways confer resistance to multiple classes	*An. arabiensis*, *Ae. aegypti*	Reduces available options for rotation
Resistance reversal	Use of synergists like PBO to restore susceptibility	*An. gambiae*, *Cx. pipiens*	Enhances efficacy of existing tools

Taxonomic note: All genus and species names are italicized in the table (e.g., Anopheles gambiae, Aedes aegypti, Culex pipiens). Genus names are written in full at first mention and abbreviated thereafter (e.g., A. gambiae).

Ace-1 = acetylcholinesterase-1 gene, CarE = carboxylesterases, GST = glutathione S-transferases, IRS = indoor residual spraying, IVM = integrated vector management, kdr = knockdown resistance, LLIN = long-lasting insecticidal nets, P450s = cytochrome P450 monooxygenases, VGSC = voltage-gated sodium channel.

## Author contributions

**Conceptualization:** Ebrahim Abbasi.

**Data curation:** Ebrahim Abbasi.

**Formal analysis:** Ebrahim Abbasi.

**Funding acquisition:** Ebrahim Abbasi.

**Investigation:** Ebrahim Abbasi.

**Methodology:** Ebrahim Abbasi.

**Project administration:** Ebrahim Abbasi.

**Resources:** Ebrahim Abbasi.

**Software:** Ebrahim Abbasi.

**Supervision:** Ebrahim Abbasi.

**Validation:** Ebrahim Abbasi.

**Visualization:** Ebrahim Abbasi.

**Writing – original draft:** Ebrahim Abbasi.

**Writing – review & editing:** Ebrahim Abbasi.

## Supplementary Material


