# Supplementary material for: Mechanisms of insecticide resistance in mosquitoes: A systematic review of biochemical and physiological perspectives for sustainable vector control
Source: Medicine (Baltimore). 2026 Mar 27;105(13):e48068. doi: 10.1097/MD.0000000000048068 (PMC13034918; doi:10.1097/MD.0000000000048068)
Supplement: Supplementary file 1 [file medi-105-e48068-s001.docx]

**Mechanisms of Insecticide Resistance in Mosquitoes: A Systematic Review of Biochemical and Physiological Perspectives for Sustainable Vector Control**

**Raw syntax**

(Disease) OR (control) OR (Disease AND control) OR (“Disease Vector”) OR (Vector AND Disease) OR (Vectors AND Disease) OR (Infectious AND Disease AND Vectors) OR (“Disease Vector” AND Infectious) OR (“Disease Vectors” AND Infectious) OR (Infectious AND Disease AND Vector) OR (Vector AND “Infectious Disease”) OR (Vectors AND “Infectious Disease”) OR (“Insect Vector”) OR (Vector AND Insect) OR (Vectors AND Insect) OR (“Mosquito Vector”) OR (Vector AND Mosquito) OR (Vectors AND Mosquito) OR (Mosquitoes) OR (Mosquitos) OR (Mosquito) OR (“Mosquito-Borne Disease”) OR (Mosquito AND Borne AND Diseases) OR (Mosquito AND Borne AND Disease) OR (“Aedes-Borne Diseases”) OR (“Aedes-Borne Disease”) OR (Aedes AND Borne AND Diseases) OR (Disease AND Aedes-Borne) OR (Mosquito-Borne AND “Viral Diseases”) OR (Disease AND “Mosquito-Borne Viral”) OR (Mosquito-Borne AND “Viral Disease”) OR (Mosquito AND Borne AND Viral AND Diseases) OR (“Viral Disease” AND Mosquito-Borne) OR (“Anopheles-Borne Diseases”) OR (“Anopheles-Borne Disease”) OR (Anopheles AND Borne AND Diseases) OR (Disease AND Anopheles-Borne) OR (Mosquito-Borne AND “Parasitic Diseases”) OR (Disease AND “Mosquito-Borne Parasitic”) OR (Mosquito-Borne AND “Parasitic Disease”) OR (Mosquito AND Borne AND Parasitic AND Diseases) OR (“Parasitic Disease” AND Mosquito-Borne) OR (Control AND Mosquito)

**PubMed/Medline**

((Disease) OR (control) OR (Disease AND control) OR (“Disease Vector”) OR (Vector AND Disease) OR (Vectors AND Disease) OR (Infectious AND Disease AND Vectors) OR (“Disease Vector” AND Infectious) OR (“Disease Vectors” AND Infectious) OR (Infectious AND Disease AND Vector) OR (Vector AND “Infectious Disease”) OR (Vectors AND “Infectious Disease”) OR (“Insect Vector”) OR (Vector AND Insect) OR (Vectors AND Insect) OR (“Mosquito Vector”) OR (Vector AND Mosquito) OR (Vectors AND Mosquito) OR (Mosquitoes) OR (Mosquitos) OR (Mosquito) OR (“Mosquito-Borne Disease”) OR (Mosquito AND Borne AND Diseases) OR (Mosquito AND Borne AND Disease) OR (“Aedes-Borne Diseases”) OR (“Aedes-Borne Disease”) OR (Aedes AND Borne AND Diseases) OR (Disease AND Aedes-Borne) OR (Mosquito-Borne AND “Viral Diseases”) OR (Disease AND “Mosquito-Borne Viral”) OR (Mosquito-Borne AND “Viral Disease”) OR (Mosquito AND Borne AND Viral AND Diseases) OR (“Viral Disease” AND Mosquito-Borne) OR (“Anopheles-Borne Diseases”) OR (“Anopheles-Borne Disease”) OR (Anopheles AND Borne AND Diseases) OR (Disease AND Anopheles-Borne) OR (Mosquito-Borne AND “Parasitic Diseases”) OR (Disease AND “Mosquito-Borne Parasitic”) OR (Mosquito-Borne AND “Parasitic Disease”) OR (Mosquito AND Borne AND Parasitic AND Diseases) OR (“Parasitic Disease” AND Mosquito-Borne) OR (Control AND Mosquito))

**Embase**

((Disease) OR (control) OR (Disease AND control) OR (‘Disease Vector’) OR (Vector AND Disease) OR (Vectors AND Disease) OR (Infectious AND Disease AND Vectors) OR (‘Disease Vector’ AND Infectious) OR (‘Disease Vectors’ AND Infectious) OR (Infectious AND Disease AND Vector) OR (Vector AND ‘Infectious Disease’) OR (Vectors AND ‘Infectious Disease’) OR (‘Insect Vector’) OR (Vector AND Insect) OR (Vectors AND Insect) OR (‘Mosquito Vector’) OR (Vector AND Mosquito) OR (Vectors AND Mosquito) OR (Mosquitoes) OR (Mosquitos) OR (Mosquito) OR (‘Mosquito-Borne Disease’) OR (Mosquito AND Borne AND Diseases) OR (Mosquito AND Borne AND Disease) OR (‘Aedes-Borne Diseases’) OR (‘Aedes-Borne Disease’) OR (Aedes AND Borne AND Diseases) OR (Disease AND Aedes-Borne) OR (Mosquito-Borne AND ‘Viral Diseases’) OR (Disease AND ‘Mosquito-Borne Viral’) OR (Mosquito-Borne AND ‘Viral Disease’) OR (Mosquito AND Borne AND Viral AND Diseases) OR (‘Viral Disease’ AND Mosquito-Borne) OR (‘Anopheles-Borne Diseases’) OR (‘Anopheles-Borne Disease’) OR (Anopheles AND Borne AND Diseases) OR (Disease AND Anopheles-Borne) OR (Mosquito-Borne AND ‘Parasitic Diseases’) OR (Disease AND ‘Mosquito-Borne Parasitic’) OR (Mosquito-Borne AND ‘Parasitic Disease’) OR (Mosquito AND Borne AND Parasitic AND Diseases) OR (‘Parasitic Disease’ AND Mosquito-Borne) OR (Control AND Mosquito))

**Scopus=**

(ALL(Disease) OR ALL(control) OR ALL(Disease AND control) OR ALL(“Disease Vector”) OR ALL(Vector AND Disease) OR ALL(Vectors AND Disease) OR ALL(Infectious AND Disease AND Vectors) OR ALL(“Disease Vector” AND Infectious) OR ALL(“Disease Vectors” AND Infectious) OR ALL(Infectious AND Disease AND Vector) OR ALL(Vector AND “Infectious Disease”) OR ALL(Vectors AND “Infectious Disease”) OR ALL(“Insect Vector”) OR ALL(Vector AND Insect) OR ALL(Vectors AND Insect) OR ALL(“Mosquito Vector”) OR ALL(Vector AND Mosquito) OR ALL(Vectors AND Mosquito) OR ALL(Mosquitoes) OR ALL(Mosquitos) OR ALL(Mosquito) OR ALL(“Mosquito-Borne Disease”) OR ALL(Mosquito AND Borne AND Diseases) OR ALL(Mosquito AND Borne AND Disease) OR ALL(“Aedes-Borne Diseases”) OR ALL(“Aedes-Borne Disease”) OR ALL(Aedes AND Borne AND Diseases) OR ALL(Disease AND Aedes-Borne) OR ALL(Mosquito-Borne AND “Viral Diseases”) OR ALL(Disease AND “Mosquito-Borne Viral”) OR ALL(Mosquito-Borne AND “Viral Disease”) OR ALL(Mosquito AND Borne AND Viral AND Diseases) OR ALL(“Viral Disease” AND Mosquito-Borne) OR ALL(“Anopheles-Borne Diseases”) OR ALL(“Anopheles-Borne Disease”) OR ALL(Anopheles AND Borne AND Diseases) OR ALL(Disease AND Anopheles-Borne) OR ALL(Mosquito-Borne AND “Parasitic Diseases”) OR ALL(Disease AND “Mosquito-Borne Parasitic”) OR ALL(Mosquito-Borne AND “Parasitic Disease”) OR ALL(Mosquito AND Borne AND Parasitic AND Diseases) OR ALL(“Parasitic Disease” AND Mosquito-Borne) OR ALL(Control AND Mosquito))

**WOS**

(ALL=(Disease) OR ALL=(control) OR ALL=(Disease AND control) OR ALL=(“Disease Vector”) OR ALL=(Vector AND Disease) OR ALL=(Vectors AND Disease) OR ALL=(Infectious AND Disease AND Vectors) OR ALL=(“Disease Vector” AND Infectious) OR ALL=(“Disease Vectors” AND Infectious) OR ALL=(Infectious AND Disease AND Vector) OR ALL=(Vector AND “Infectious Disease”) OR ALL=(Vectors AND “Infectious Disease”) OR ALL=(“Insect Vector”) OR ALL=(Vector AND Insect) OR ALL=(Vectors AND Insect) OR ALL=(“Mosquito Vector”) OR ALL=(Vector AND Mosquito) OR ALL=(Vectors AND Mosquito) OR ALL=(Mosquitoes) OR ALL=(Mosquitos) OR ALL=(Mosquito) OR ALL=(“Mosquito-Borne Disease”) OR ALL=(Mosquito AND Borne AND Diseases) OR ALL=(Mosquito AND Borne AND Disease) OR ALL=(“Aedes-Borne Diseases”) OR ALL=(“Aedes-Borne Disease”) OR ALL=(Aedes AND Borne AND Diseases) OR ALL=(Disease AND Aedes-Borne) OR ALL=(Mosquito-Borne AND “Viral Diseases”) OR ALL=(Disease AND “Mosquito-Borne Viral”) OR ALL=(Mosquito-Borne AND “Viral Disease”) OR ALL=(Mosquito AND Borne AND Viral AND Diseases) OR ALL=(“Viral Disease” AND Mosquito-Borne) OR ALL=(“Anopheles-Borne Diseases”) OR ALL=(“Anopheles-Borne Disease”) OR ALL=(Anopheles AND Borne AND Diseases) OR ALL=(Disease AND Anopheles-Borne) OR ALL=(Mosquito-Borne AND “Parasitic Diseases”) OR ALL=(Disease AND “Mosquito-Borne Parasitic”) OR ALL=(Mosquito-Borne AND “Parasitic Disease”) OR ALL=(Mosquito AND Borne AND Parasitic AND Diseases) OR ALL=(“Parasitic Disease” AND Mosquito-Borne) OR ALL=(Control AND Mosquito))

**ProQuest**

(ALL,FT(Disease) OR ALL,FT(control) OR ALL,FT(Disease AND control) OR ALL,FT(“Disease Vector”) OR ALL,FT(Vector AND Disease) OR ALL,FT(Vectors AND Disease) OR ALL,FT(Infectious AND Disease AND Vectors) OR ALL,FT(“Disease Vector” AND Infectious) OR ALL,FT(“Disease Vectors” AND Infectious) OR ALL,FT(Infectious AND Disease AND Vector) OR ALL,FT(Vector AND “Infectious Disease”) OR ALL,FT(Vectors AND “Infectious Disease”) OR ALL,FT(“Insect Vector”) OR ALL,FT(Vector AND Insect) OR ALL,FT(Vectors AND Insect) OR ALL,FT(“Mosquito Vector”) OR ALL,FT(Vector AND Mosquito) OR ALL,FT(Vectors AND Mosquito) OR ALL,FT(Mosquitoes) OR ALL,FT(Mosquitos) OR ALL,FT(Mosquito) OR ALL,FT(“Mosquito-Borne Disease”) OR ALL,FT(Mosquito AND Borne AND Diseases) OR ALL,FT(Mosquito AND Borne AND Disease) OR ALL,FT(“Aedes-Borne Diseases”) OR ALL,FT(“Aedes-Borne Disease”) OR ALL,FT(Aedes AND Borne AND Diseases) OR ALL,FT(Disease AND Aedes-Borne) OR ALL,FT(Mosquito-Borne AND “Viral Diseases”) OR ALL,FT(Disease AND “Mosquito-Borne Viral”) OR ALL,FT(Mosquito-Borne AND “Viral Disease”) OR ALL,FT(Mosquito AND Borne AND Viral AND Diseases) OR ALL,FT(“Viral Disease” AND Mosquito-Borne) OR ALL,FT(“Anopheles-Borne Diseases”) OR ALL,FT(“Anopheles-Borne Disease”) OR ALL,FT(Anopheles AND Borne AND Diseases) OR ALL,FT(Disease AND Anopheles-Borne) OR ALL,FT(Mosquito-Borne AND “Parasitic Diseases”) OR ALL,FT(Disease AND “Mosquito-Borne Parasitic”) OR ALL,FT(Mosquito-Borne AND “Parasitic Disease”) OR ALL,FT(Mosquito AND Borne AND Parasitic AND Diseases) OR ALL,FT(“Parasitic Disease” AND Mosquito-Borne) OR ALL,FT(Control AND Mosquito))

**BioOne**

((Disease) OR (control) OR (Disease AND control) OR (“Disease Vector”) OR (Vector AND Disease) OR (Vectors AND Disease) OR (Infectious AND Disease AND Vectors) OR (“Disease Vector” AND Infectious) OR (“Disease Vectors” AND Infectious) OR (Infectious AND Disease AND Vector) OR (Vector AND “Infectious Disease”) OR (Vectors AND “Infectious Disease”) OR (“Insect Vector”) OR (Vector AND Insect) OR (Vectors AND Insect) OR (“Mosquito Vector”) OR (Vector AND Mosquito) OR (Vectors AND Mosquito) OR (Mosquitoes) OR (Mosquitos) OR (Mosquito) OR (“Mosquito-Borne Disease”) OR (Mosquito AND Borne AND Diseases) OR (Mosquito AND Borne AND Disease) OR (“Aedes-Borne Diseases”) OR (“Aedes-Borne Disease”) OR (Aedes AND Borne AND Diseases) OR (Disease AND Aedes-Borne) OR (Mosquito-Borne AND “Viral Diseases”) OR (Disease AND “Mosquito-Borne Viral”) OR (Mosquito-Borne AND “Viral Disease”) OR (Mosquito AND Borne AND Viral AND Diseases) OR (“Viral Disease” AND Mosquito-Borne) OR (“Anopheles-Borne Diseases”) OR (“Anopheles-Borne Disease”) OR (Anopheles AND Borne AND Diseases) OR (Disease AND Anopheles-Borne) OR (Mosquito-Borne AND “Parasitic Diseases”) OR (Disease AND “Mosquito-Borne Parasitic”) OR (Mosquito-Borne AND “Parasitic Disease”) OR (Mosquito AND Borne AND Parasitic AND Diseases) OR (“Parasitic Disease” AND Mosquito-Borne) OR (Control AND Mosquito))

***Availability of Data and Materials***

All data generated or analyzed during this study are included in this published article and its supplementary materials. The full search strategies and Boolean search strings used for each database (PubMed, Web of Science, and Scopus) are provided in Supplementary File 1, enabling the complete reproducibility of the literature search.
